# FOREWORD

**DOI:** 10.2174/138920212799034758

**Published:** 2012-03

**Authors:** Christian Neri

The past year has seen several important developments for *Current Genomics.*

We have achieved a citation index impact factor of 2.48, indicating that the Journal reaches an increasingly wide audience. To achieve a citation index, papers must be formatted by the publisher and submitted for indexing. I want to thank the staff of Bentham Science for their effort in handling these procedures and ensuring the journal reaches a large audience. I also want to thank the members of the Editorial Board, the Section Editors, Associate Editors and Co-Editor for their effort in promoting editorial diversity and excellence in the wide field of genome sciences.

*Current Genomics* aims at providing a unique forum for discussing the impact of genome sciences and systems biology in specific fields of research such as Evolution, Developmental biology, Aging and Disease research. To this end, the journal offers different formats such as regular reviews as well as Mini-Hot-Topic and Hot-Topic issues.

This past year, we published five Hot-Topics issues including ‘Genes Coding Industrially Relevant Enzymes in Fungi: Isolation and Protein Engineering of Laccases’ edited by Dr. Vincenza Faraco, ‘Genomics of Childhood Obesity’ edited by Dr. Merlin G. Butler, ‘Retinitis pigmentosa: criticisms and management’ edited by Dr. Francesco Parmeggiani, ‘Evolutionary system biology’ edited by Dr. Jianying Gu, and ‘Thyroid: from genes to the disease’ edited by Dr. Katja Zaletel. All five issues greatly illustrate the increasingly important role of genome sciences in the various fields of biology and medicine.

We plan to publish five more Hot-Topics issues this year including ‘Replicating strand asymmetry in bacterial and eukaryotic genomes’ organized by Dr. Feng-Biao Guo, ‘Comparative genomics and genome evolution’ organized by Drs. Sabyasachi Das and Masayuki Hirano, ‘Genetics dissection of complex traits in the genomic era’ organized by Dr. Bernardo Ordas, ‘Genomics-based medicine, single cell analysis and the prospects for risk assessment’ organized by Dr. H.-Ulli Weier and ‘Genomic approaches to aging’ organized by Dr. Matt Kaeberlein.

As always, we welcome suggestions for special Hot-Topic and Mini-Hot-Topic issues and guest editors to organize them. At the same time, we will continue to consider submissions of comprehensive and authoritarive regular reviews on topics of interest to a broad readership in the field of genome science and beyond.

On behalf of the Editorial Board, I would like to thank all the contributors to the volumes of *Current Genomics*, and I am looking forward to working with many new authors in the future.

